# SARS-CoV-2 Co-Infections and Recombinations Identified by Long-Read Single-Molecule Real-Time Sequencing

**DOI:** 10.1128/spectrum.00493-23

**Published:** 2023-06-01

**Authors:** Pauline Trémeaux, Justine Latour, Noémie Ranger, Vénicia Ferrer, Agnès Harter, Romain Carcenac, Pauline Boyer, Sofia Demmou, Florence Nicot, Stéphanie Raymond, Jacques Izopet

**Affiliations:** a Virology Laboratory, Toulouse University Hospital, Toulouse, France; b INSERM UMR 1291 – CNRS UMR 5051, Toulouse Institute for Infectious and Inflammatory Diseases (INFINITy), Toulouse, France; Kumamoto Daigaku

**Keywords:** SARS-CoV-2, co-infection, genomic surveillance, long-read sequencing, quasispecies, recombination

## Abstract

Co-infection with at least 2 strains of virus is the prerequisite for recombination, one of the means of genetic diversification. Little is known about the prevalence of these events in SARS-CoV-2, partly because it is difficult to detect them. We used long-read PacBio single-molecule real-time (SMRT) sequencing technology to sequence whole genomes and targeted regions for haplotyping. We identified 17 co-infections with SARS-CoV-2 strains belonging to different clades in 6829 samples sequenced between January and October, 2022 (prevalence 0.25%). There were 3 Delta/Omicron co-infections and 14 Omicron/Omicron co-infections (4 cases of 21K/21L, 1 case of 21L/22A, 2 cases of 21L/22B, 4 cases of 22A/22B, 2 cases of 22B/22C and 1 case of 22B/22E). Four of these patients (24%) also harbored recombinant minor haplotypes, including one with a recombinant virus that was selected in the viral quasispecies over the course of his chronic infection. While co-infections remain rare among SARS-CoV-2-infected individuals, long-read SMRT sequencing is a useful tool for detecting them as well as recombinant events, providing the basis for assessing their clinical impact, and a precise indicator of epidemic evolution.

**IMPORTANCE** SARS-CoV-2 variants have been responsible for the successive waves of infection over the 3 years of pandemic. While co-infection followed by recombination is one driver of virus evolution, there have been few reports of co-infections, mainly between Delta and Omicron variants or between the first 2 Omicron variants 21K_BA.1 and 21L_BA.2. The 17 co-infections we detected during 2022 included cases with the recent clades of Omicron 22A, 22B, 22C, and 22E; 24% harbored recombinant variants. This study shows that long-read SMRT sequencing is well suited to SARS-CoV-2 genomic surveillance.

## INTRODUCTION

The Severe Acute Respiratory Syndrome Coronavirus 2 (SARS-CoV-2) that caused the COVID-19 pandemic has evolved genetically since its appearance in late 2019, resulting in a succession of variants. Two nomenclature systems are used to define SARS-CoV-2 strains: Nextstrain clades (1) and Pangolin lineages (2). The World Health Organization (WHO) has also defined: Variants of Interest (VOIs) - strains harboring specific mutations that could influence virus transmissibility or disease severity and whose prevalence increases, and Variants of Concern (VOCs) - strains that also influence global public health (3). These variants are identified by Greek letters. This succession of variants, plus changes in public health measures, partially explains the successive waves of SARS-CoV-2 infections. For example, the VOC Delta 21A/I/J (B.1.617.2 and sub-lineages) was progressively replaced by the VOC Omicron in France during the wave of December 2021 to February 2022. The initial Omicron strain then evolved into various clades/sub-lineages: 21K_BA.1 and BA.1.1 were replaced by 21L_BA.2, mostly responsible for the March-April 2022 wave. Strains 22A_BA.4 and 22B_BA.5 appeared later and were predominant during the late June-July 2022 wave (4, 5). SARS-CoV-2 22E_BQ.1 and its sub-lineages evolved from the 22B_BA.5 lineage at the end of 2022 and became predominant during the most recent November-December 2022 wave (6).

Many countries have set up systems to monitor the genetic evolution of circulating SARS-CoV-2 strains, that involve detecting variant-specific mutations, and/or sequencing randomly selected samples. In 2022, the French authorities have recommended targeted molecular screening of positive samples for mutations in the S gene: E484K, L452R, and one among K417N, del69/70, S371L-S373P, Q493R, to discriminate between the VOCs Delta and Omicron (7, 8).

The co-existence of several strains, especially during periods of high viral circulation, provides the basis for SARS-CoV-2 co-infections and hence potential recombination. Most of the reported cases of co-infections in various countries have been either Delta/Omicron co-infections (9–13), or, more recently, co-infections with the Omicron sub-lineages 21K_BA.1/21L_BA.2 (14, 15). Recombinant viruses, such as the so-called “Deltacron” have also been reported (9, 16), and are designated in the Pangolin taxonomy by lineages starting with “X”.

PacBio single-molecule real-time (SMRT) sequencing provides long reads suitable for both accurate whole genome and S gene quasispecies analyses (17–19). This study uses SMRT sequencing to analyze the prevalence of co-infections with two clades of SARS-CoV-2 and explore their potential for recombination.

## RESULTS

### Prevalence of SARS-CoV-2 co-infection in South-West France; 2022.

**FIG 1:**
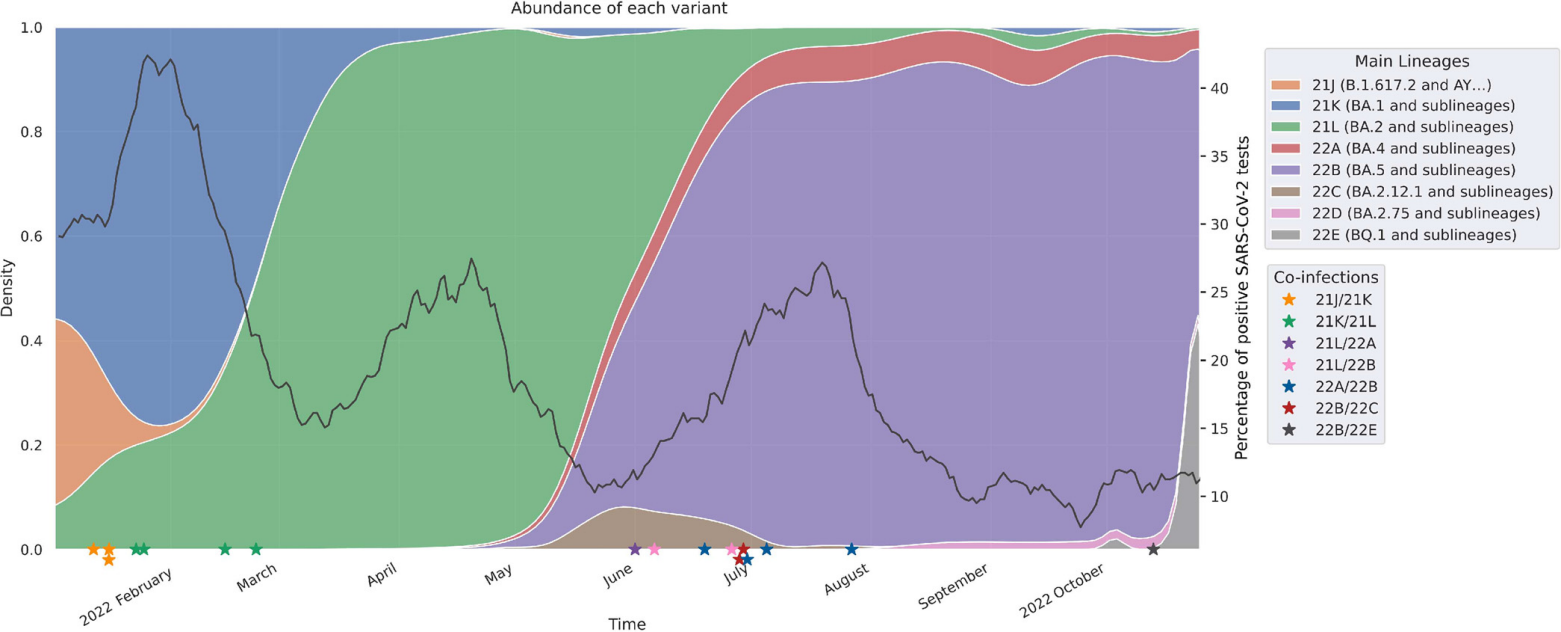
SARS-CoV-2 lineages in France in 2022. Colored areas show the relative abundance of each SARS-CoV-2 clade, calculated from our sequencing results (left axis). The black line represents the percentage of positive SARS-CoV-2 tests (7-day moving average), illustrating the successive waves of infection (right axis). Stars on the *x* axis indicate the 17 cases of co-infections.

We suspected a co-infection when more than 10 nucleotide positions over the complete genome were occupied by more than 1 nucleotide base, with a relative abundance of 10 to 90%. Every case of co-infection was confirmed with a second nucleic acid extraction: the sequences of both the full-length SARS-CoV-2 genome and the region encoding the Spike S1 domain were determined for each of the 2 extracts. From the 6829 samples sequenced between January and October 2022, we identified 17 individuals (23 samples) co-infected with strains from different clades, for an overall co-infection frequency of about 0.25%. There were 3 Delta/Omicron (21J/21K) co-infections and 14 Omicron/Omicron co-infections: 4 cases of 21K/21L, 1 case of 21L/22A, 2 cases of 21L/22B, 4 cases of 22A/22B, 2 cases of 22B/22C and 1 case of 22B/22E co-infection (Fig. 1). The co-infections peaked during 2 periods, winter and summer of 2022, when both the SARS-CoV-2 circulation was high (5th and 7th waves of infection in France) and involved different clades (Fig. 1). In contrast, we found no case during the spring months, despite the 6th wave of infection, which consisted almost exclusively of the Omicron 21L_BA.2 variant, or during the marked predominance of the 22B_BA.5 variant, which occurred later. The most recent case of co-infection occurred after the emergence of the 22E_BQ.1 strain.

We also found 4 cases of infection with a recombinant virus. As neither of the parental strains were detected, they probably resulted from the transmission of already recombinant viruses. We identified 1 case of XAF recombinant (BA.1/BA.2), 1 case of XAZ recombinant (BA.2.75/BA.5), and 1 case of XBB.1 recombinant (BA.2.75/BJ.1). The fourth case was a BA.1/BA.2 recombinant strain without a Pangolin lineage name, but similar mutation profiles have been reported (https://github.com/cov-lineages/pango-designation/issues/514). The overall estimated frequency of infection with a recombinant strain was about 0.06%.

The gender distribution of co-infections was 41% male and 59% female, with a median age of 41 (range: 20 to 72). These demographic data are not significantly different from those of individuals with no co-infection (48% males, *P-value *= 0.73; median age 38 (range: 0 to 101 years), *P-value *= 0.44). Most (14/17) of the co-infection cases identified came to our testing center: 9/14 had received 3 doses of anti-SARS-CoV-2 vaccine, 1/14 had received only 2 doses, and the vaccination status of the other 4 was unknown. The last 3 cases were identified in our hospital: Patient 1 was admitted to the intensive care unit (ICU) for a severe infection (no vaccination) and the other 2 were immunocompromised. One of them was treated with Sotrovimab, an anti-SARS-CoV-2 monoclonal antibody (Table 1, Patient 7).

**TABLE 1:** Demographic data of SARS-CoV-2 co-infected patients

Patient #	Sex	Age	Context	Vaccination status	Sample	Sample date	Mutations screening^*a*^	Ct
1	M	72	Hospitalization in ICU^*b*^ (heart failure, BMI^*c*^ 26.8). Viremic (Ct = 30). Deceased	No vaccination	Bronchoalveolar lavage fluid	12/01/22	K417N + L452R	17.3
2	F	47	Testing center	3 doses Pfizer	Nasopharyngeal swab	16/01/22	K417N + L452R SGTL (ΔCt = 3)	16.5
3	F	53	Testing center	2 doses Moderna	Nasopharyngeal swab	16/01/22	K417N + L452R SGTL (ΔCt = 7)	13.3
4	F	23	Testing center	3 doses Pfizer	Nasopharyngeal swab	23/01/22	K417N (No L452R) SGTL (ΔCt = 3)	13.3
5	F	20	Testing center	3 doses Moderna	Nasopharyngeal swab	25/01/22	K417N (No L452R) Non-SGTF/SGTL	18.1
6	F	42	Testing center	3 doses Moderna	Nasopharyngeal swab	15/02/22	K417N (No L452R) Non-SGTF/SGTL	19.2
7	M	52	Immunodepression (chemotherapy against myeloma). D^*d*^20 Sotrovimab	3 doses Pfizer (with no Ab response)	Nasopharyngeal swab	23/02/22	Non-SGTF/SGTL	18.9
8	M	21	Immunodepression (first chemotherapy against testicular carcinoma)	2 doses Pfizer + 1 previous infection (Jan, 2022)	Nasopharyngeal swab	01/06/2022	K417N + L452R	15.3
9	M	47	Testing center	3 doses Pfizer	Nasopharyngeal swab	06/06/2022	K417N + L452R	15.2
10	F	41	Testing center	3 doses Pfizer	Nasopharyngeal swab	19/06/2022	K417N + L452R	17.6
11	M	66	Testing center	3 doses Pfizer	Nasopharyngeal swab	26/06/2022	K417N + L452R	18.8
12	F	23	Testing center	unknown	Nasopharyngeal swab	28/06/2022	K417N + L452R	19.5
13	M	27	Testing center	3 doses Pfizer	Nasopharyngeal swab	29/06/2022	K417N + L452R	18.5
14	F	28	Testing center	3 doses Pfizer	Nasopharyngeal swab	30/06/2022	K417N + L452R	10.2
15	F	26	Testing center	unknown	Nasopharyngeal swab	05/07/2022	K417N + L452R	15.2
16	F	26	Testing center	unknown	Nasopharyngeal swab	22/07/2022	K417N + L452R	15.7
17	M	46	Testing center	unknown	Nasopharyngeal swab	13/10/2022	K417N + L452R	17.9

a Screened using the ID SARS-CoV-2/VOCs Revolution Pentaplex assay (K417N and L452R mutations) and/or the ThermoFisher TaqPath RT-PCR assay (SGTF: S gene target failure; SGTL: S gene target late detection. Both are consistent with the presence of del69/70).

b ICU: Intensive Care Unit.

c BMI: Body Mass Index.

d D: day.

### Co-infection viral characteristics.

**FIG 2:**
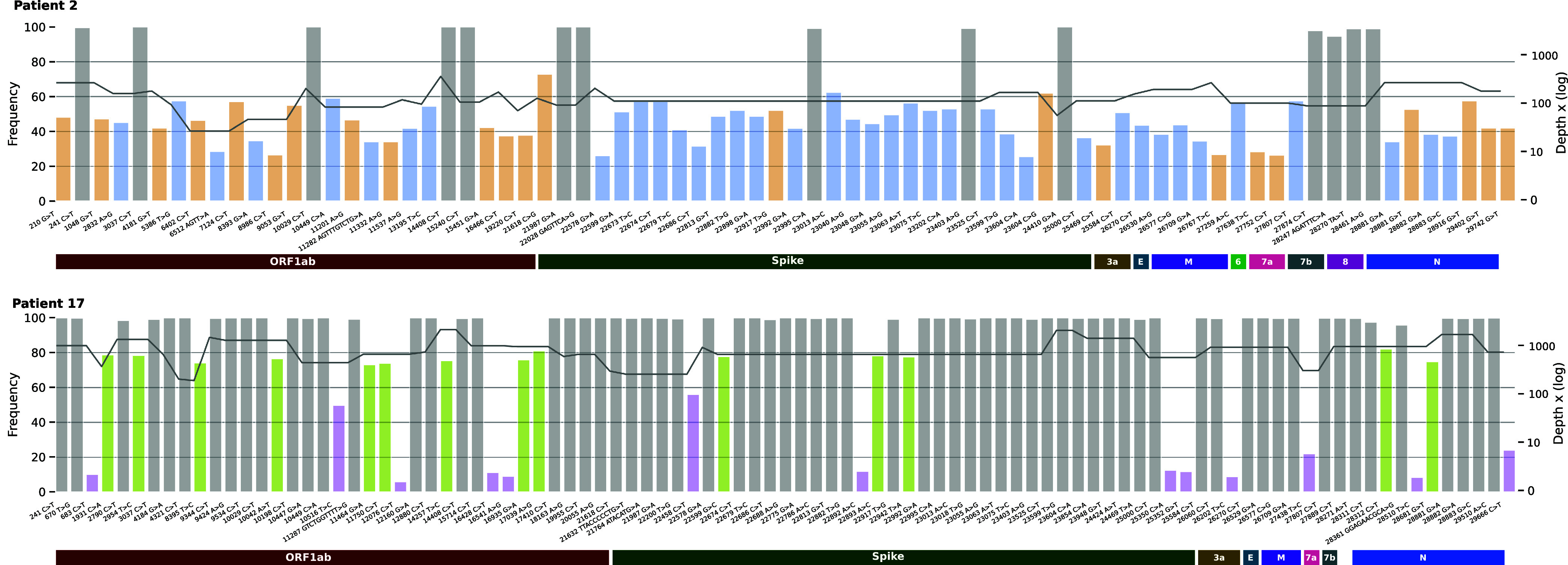
Nucleotide mutation frequencies on the complete genome. Patient 2 (top panel) and Patient 17 (bottom panel): relative abundance of mutations along the whole genome, compared to the Wuhan reference sequence (left axis). Gray bars represent polymorphisms common to the two infecting SARS-CoV-2 lineages and colored bars represent clade/lineage-defining polymorphisms (yellow: 21A/I/J_B.1.617.2, blue: 21K_BA.1, purple: 22B_BA.5, light green: 22E_BQ.1). Black lines show the coverage depth (right axis).

We determined the relative abundance of all mutations specific to only 1 of the 2 strains present in each sample from the SARS-CoV-2 complete genome variant calling results. The abundance of each strain of a co-infection was estimated by the mean abundance value of its specific mutations. The relative abundance of the minor viral strains varied from 15% to 50% (Table 2, Fig. 2 and Fig. S1). Duplicate analyses of full genome sequences provided similar results.

**TABLE 2:** Characteristics of SARS-CoV-2 co-infections

Patient #	Clades	Lineages	Relative abundance^*a*^	2.5 kb haplotypes^*b*^	GISAID
1	21J/21K	B.1.617.2/BA.1.1	50%/50%	B.1.617.2 (52%) / BA.1.1 (48%)	EPI_ISL_16507432
2	21J/21K	B.1.617.2/BA.1.1	50%/50%	B.1.617.2 (57%) / BA.1.1 (43%)	EPI_ISL_16507433
3	21J/21K	AY.4/BA.1.1	75%/25%	AY.4 (72%) / BA.1.1 (28%)	EPI_ISL_16507434
4	21K/21L	BA.1.1/BA.2	70%/30%	BA.1.1 (83%) / BA.2 (17%)	EPI_ISL_16507435
5	21K/21L	BA.1/BA.2	25%/75%	BA.1 (16%) / BA.2 (84%)	EPI_ISL_16507436
6	21K/21L	BA.1.1/BA.2	50%/50%	BA.1.1 (45%) / BA.2 (55%)	EPI_ISL_16507437
7	21K/21L	BA.1.1/BA.2	35%/65%	BA.1.1 (40%) / BA.2 + E340A (60%)	EPI_ISL_16507438
8	21L/22A	BA.2/BA.4	25%/75%	BA.2 + L452M (12%) BA.4 (66%) recombinant BA.4/BA.2 (L452M) (12%) recombinant BA.2/BA.4 (10%)	EPI_ISL_13502102
9	21L/22B	BA.2/BA.5	35%/65%	BA.2 (41%) / BA.5 (59%)	EPI_ISL_16507439
10	22A/22B	BA.4/BA.5	15%/85%	BA.4 and/or BA.5 (100%)	EPI_ISL_13950692
11	21L/22B	BA.2/BA.5	25%/75%	BA.2 (24%) / BA.5 (76%)	EPI_ISL_13950697
12	22B/22C	BA.5/BA.2.12.1	35%/65%	BA.2.12.1 (58%) BA.5 (20%) recombinant BA.2.12.1/ BA.5 (L452Q) (9%) recombinant BA.2.12.1/ BA.5 (L452R) (6%) recombinant BA.5/BA.2.12.1 (7%)	EPI_ISL_13974755
13	22B/22C	BA.5/BA.2.12.1	60%/40%	BA.2.12.1 (46%) BA.5 (37%) recombinant BA.2.12.1/ BA.5 (12%) recombinant BA.5/BA.2.12.1 (5%)	EPI_ISL_16507440
14	22A/22B	BA.4/BA.5	50%/50%	BA.4 (63%) / BA.5 (37%)	EPI_ISL_14232543
15	22A/22B	BA.4.6/BA.5	80%/20%	Inconclusive	EPI_ISL_14232544
16	22A/22B	BA.4.4/BA.5.1	20%/80%	BA.4 (19%) / BA.5 (81%)	EPI_ISL_14497373
17	22B/22E	BA.5.1/BQ.1.1	25%/75%	BA.5.1 wild type (9%) BA.5.1 + K444Q (12%) BQ.1.1 (79%)	EPI_ISL_15777849

a Relative abundance was calculated from the variant calling results throughout the whole SARS-CoV-2 genome: the abundance of each strain is the mean of the prevalence values of mutations specific to its lineage.

b Based on S gene sequencing. The abundance of each haplotype was calculated as the number of reads of this haplotype/total number of reads of the sample.

We also analyzed the proportion of each strain in the SARS-CoV-2 quasispecies by haplotyping a 2.5-kb amplicon on the S gene; encoding the Spike protein S1 domain. Haplotypes harboring frameshifts were considered to be PCR errors and discarded. Minor haplotypes accounting for over 5%, confirmed on 2 distinct extractions/amplifications, were included. There were usually 2 haplotypes corresponding to the strains identified by complete genome analysis, with concordant abundances (Table 2). Since many 22A_BA.4 and 22B_BA.5 strains have similar S gene sequences, we could not always identify them using the Spike amplicon analysis. A single haplotype was observed in Patient 10, harboring a 22A_BA.4/22B_BA.5 co-infection. The Spike amplicon analysis for Patient 15 was considered inconclusive, while Patient 17 harbored an additional haplotype corresponding to a 22B_BA.5.1 strain that had acquired a K444Q mutation on the S gene (Table 2), reflecting intra-host virus evolution.

### SARS-CoV-2 recombination in co-infected individuals.

Haplotyping the 2.5-kb Spike amplicon also enabled us to analyze the presence of SARS-CoV-2 recombination events in co-infected patients. We identified minor recombinant haplotype(s) in addition to the 2 parental strains in 4/17 (24%) individuals, confirmed by amplification and sequencing analyses from 2 independent nucleic acid extracts (Tables 2 and 3).

**TABLE 3:** Viral evolution of a chronic infection in an immunocompromised patient^*a*^

#	Context	Sample date	Mutations screening^*b*^	Ct	Clade(s)	Lineage(s)	Relative abundance^*c*^	2.5 kb haplotypes	GISAID
7a	D0 Sotrovimab	03/02/2022	K417N (No L452R) Non-SGTF/SGTL	9.5	21L	BA.2	100%	BA.2 wt^*d*^ (100%)	EPI_ISL_10312843
7b	D4 Sotrovimab	07/02/2022	Non-SGTF/SGTL	21.0	21L	BA.2	100% (E340A)	BA.2 wt (7%) BA.2 + E340A (78%) BA.2 + E340D (15%)	EPI_ISL_10317798
7c	D7 Sotrovimab	10/02/2022	Non-SGTF/SGTL	13.5	21L	BA.2	100% (E340A)	BA.2 wt (3%) BA.2 + E340A (72%) BA.2 + E340D (15%) BA.2 + E340K (10%)	EPI_ISL_10318645
7d	D14 Sotrovimab	17/02/22	Non-SGTF/SGTL	14.5	21L	BA.2	65% E340A / 35% E340D	BA.2 wt (3%) BA.2 + E340A (70%) BA.2 + E340D (30%)	EPI_ISL_11064638
7e^*e*^	D20 Sotrovimab	23/02/22	Non-SGTF/SGTL	18.9	21K/21L	BA.1.1/BA.2	35%/65%	BA.1.1 (40%) BA.2 + E340A (60%)	EPI_ISL_16507438
7f	D22 Sotrovimab	25/02/22	K417N (No L452R) SGTL (ΔCt = 4)	17.4	21K/21L	BA.1.1/BA.2	90%/10%	BA.1.1 (89%) BA.1.1 + E340A (9%) BA.2 + E340A (2%)	EPI_ISL_17401992
7g	D27 Sotrovimab	02/03/22	K417N (No L452R) Non-SGTF/SGTL	20.7	Low coverage on the full-length genome (66%)			BA.2 + E340A + D405wt (27%) Rec.^*f*^BA.1.1/BA.2 (34%) Rec. BA.1.1/BA.2 (E340A) (34%) Rec. BA.1.1/BA.2 (E340Q) (5%)	
7h	D34 Sotrovimab	09/03/2022	SGTF	27	Low coverage on the full-length genome (36%)			Rec. BA.1.1/BA.2 (E340Q) (100%)	
7i	D41 Sotrovimab	16/03/2022	SGTF	28	Amplification failure			Rec. BA.1.1/BA.2 (E340Q) (86%) Rec. BA.1.1/BA.2 (E340Q + P491L) (14%)	
7j	D41 Sotrovimab	16/03/2022	SGTF	25	Low coverage on the full-length genome (39%)			Rec. BA.1.1/BA.2 (E340K) (56%) Rec. BA.1.1/BA.2 (E340A) (44%)	
7k	D48 Sotrovimab	23/03/2022	K417N (No L452R) SGTF	23.6	Low coverage on the full-length genome (63%)			BA.1.1 (91%) Rec. BA.1.1/BA.2 (E340Q) (9%)	
7l	D53 Sotrovimab	28/03/2022	SGTF	19.3	21K/21L	BA.1.1/BA.2 (recombinant)	100%	Rec. BA.1.1/BA.2 (E340Q) (100%)	EPI_ISL_17401993

a All samples were nasopharyngeal swabs.

b Screened using the ID SARS-CoV-2/VOCs Revolution Pentaplex assay (for K417N and L452R mutations) and/or the ThermoFisher TaqPath RT-PCR assay (SGTF: S gene target failure; SGTL: S gene target late detection. Both are consistent with the presence of del69/70).

c Relative abundance was calculated from the variant calling results throughout the whole SARS-CoV-2 genome: the abundance of each strain is the mean of the prevalence values of mutations specific to its lineage. For the 2.5 kb haplotypes, abundance was calculated as the number of reads of this haplotype/total number of reads of the sample.

d wt: wild type.

e 7e, the first sample showing a co-infection for this patient, is also included in Tables 1 and 2.

f Rec.: recombinant.

**FIG 3:**
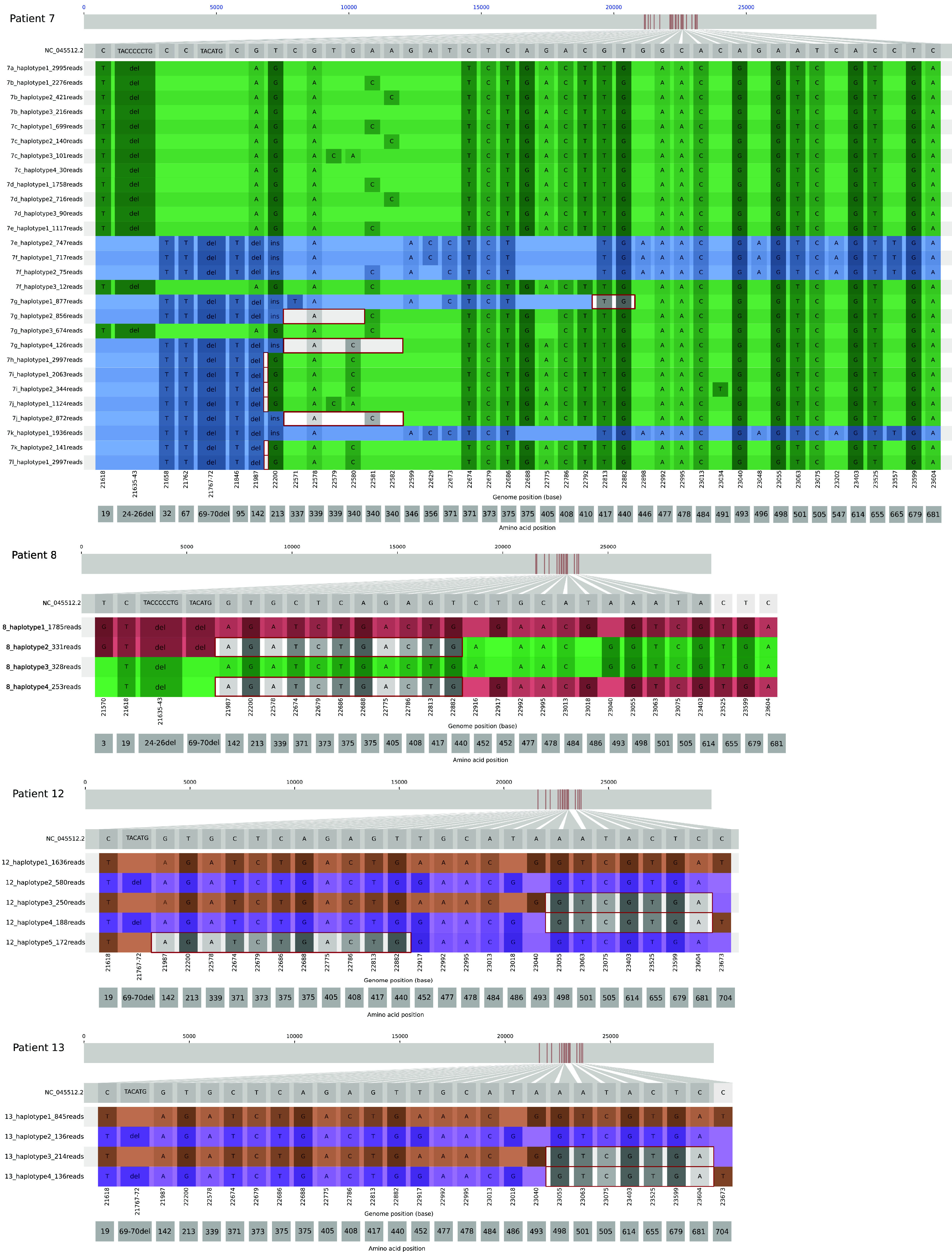
Spike amplicon haplotypes for the 4 patients with recombinant strains. Plots show nucleotide positions that differ from the Wuhan reference on the Spike A6 amplicon (genome positions: 21563-23823). Colors highlight the different clades/lineages (blue: 21K_BA.1, green: 21L_BA.2, red: 22A_BA.4, purple: 22B_BA.5, maroon: 22C_BA.1.12.1). The red boxes indicate the recombination regions. The number of reads indicated for each haplotype is the result of 1of the 2 (concordant) sequencing performed.

The 21L_BA.2 virus in Patient 8 had acquired an L452M mutation on the S gene and had recombined with a 22A_BA.4 virus to create 2 different recombinant viruses. The first recombinant, relative abundance 12%, had an S gene whose 5′ segment belonged to the 22A_BA.4 lineage plus a 3′ segment that belonged to the 21L_BA.2 lineage. The sequence of the second recombinant, abundance 10%, was the reverse (Table 2 and Fig. 3). A sample taken from this patient 7 days earlier revealed a co-infection with wild type 21L_ BA.2 (9%) and 22A_BA.4 (91%) strains but no recombinant haplotype (Fig. S2).

Patients 12 and 13 were both co-infected with 22B_BA.5 and 22C_BA.2.12.1 viruses; Patient 12 harbored 3 recombinant haplotypes in addition to the parental strains while Patient 13 harbored only 2. Patient 12 had two recombinant haplotypes with 22C_BA.2.12.1 lineage 5′ S gene segments and 22B_BA.5 lineage 3′ segments: one had a recombinant point after the mutation S:L452Q (abundance: 9%) and the other before the mutation S:L452R (abundance: 6%). The last recombinant haplotype began with the 22B_BA.5-like sequence, followed by the 22C_BA.2.12.1-like sequence (abundance: 7%). The 2 recombinant haplotypes in Patient 13 were similar to 2 of those harbored by Patient 12 (Table 2 and Fig. 3). This similarity could be due to an epidemiological link between these patients, who were living together.

Patient 7 was immunocompromised because of anti-myeloma chemotherapy and, being at risk of severe infection, was being treated with Sotrovimab. As part of this treatment protocol, nasopharyngeal swabs were regularly taken to follow the evolution of his viral load, assessed by the PCR Ct value, and to monitor the genetic evolution of his viral strain, i.e., selection of resistance mutations (Table 3). He was initially infected with a 21L_BA.2 strain (sample 7a) that acquired several mutations at S gene position E340 as early as day 4 of treatment: E340A, E340D, and E340K (samples 7b, 7c, and 7d). He became superinfected with a 21K_BA1.1 strain on day 20 (sample 7e); this rapidly became predominant and acquired an S:E340A mutation (day 22, sample 7f). Several recombinant haplotypes emerged after a week of co-infection: all had an S gene 5′ segment similar to the 21K_BA.1.1 lineage and a 3′ segment similar to the 21L_BA.2 strain, but with different break points and different parental strains (Fig. 3 and Fig. S2). The complex evolution of the SARS-CoV-2 quasispecies in this patient resulted in one predominant recombinant strain, harboring the S:E340Q mutation.

## DISCUSSION

The succession of several SARS-CoV-2 variants and the high circulation rate of the virus provided ideal conditions for co-infection. This long-read sequencing study identified co-infections with the various SARS-CoV-2 clades circulating in France in 2022, including those of the Omicron variant. The overall prevalence of co-infections with viruses of different clades was 0.25%, while 24% of co-infected individuals harbored recombinant viruses.

This co-infection rate is probably a minimum value, since some suspected cases could not be confirmed. We also identified 8 other cases of possible co-infection with lineages belonging to the same clade during this period (January to October 2022): 6 cases of 21K_BA.1/BA.1.1 infection and 2 cases of 22B infection (BE.1/BF.5 and BA.5.1/BA.5.3), which would bring the co-infection prevalence to 0.37%. However, these cases may result from the acquisition of new mutation(s) rather than co-infections, given the limited difference between the 2 strains: BA.1 and BA.1.1 lineages in particular differ only because of the S:R346K mutation.

Our prevalence results agree well with those of other French studies that used short-read sequencing (0.18-0.26% co-infections) (9, 13). However, co-infection rates vary considerably: two American studies found rates of < 0.1% and 0.37 to 0.61% (20, 21), while other reported rates were 0.61% in Brazil (22) and 5% in the United Arab Emirates (23). This might be due to epidemiologic differences between countries, as co-infections seem to occur when both SARS-CoV-2 circulation rate is high, and more than 1 variant is present. Nevertheless, there may well be co-infections with the same lineage, which would not be detected, during periods of high SARS-CoV-2 circulation with essentially a single clade, such as 21L_BA.2 in spring 2022 and 22E_BA.5 in autumn 2022 in France.

Most SARS-CoV-2 complete genome amplification and sequencing techniques rely on tiled amplicons. A few updates of the most commonly used Artic and Midnight primer sets have been published (24, 25). We too have modified the sequence of several primers. However, the ongoing genetic evolution of SARS-CoV-2 can result in mismatches between some primers and the virus genome, so that a new lineage would not be detected or have a lower coverage. The relative abundance of each lineage in a co-infection is estimated from the complete genome sequence data by the mean abundance of the mutations specific to this lineage, and might therefore be biased. This is why we used 2 complementary approaches, full-length genome sequencing and targeted sequencing of a 2.5 kb amplicon without fragmentation, to sequence SARS-CoV-2 and study co-infections. Targeted sequencing provides greater sequencing depth, and the use of long amplicons enables haplotyping. We sequenced the amplicon covering the Spike protein S1 domain because it is one of the most variable genomic regions of SARS-CoV-2, under the selective pressure of humoral responses and interactions with the cell entry receptor.

Although we have previously shown that we can accurately identify > 90% of the lineages from this region (18) it is still not always possible to identify precisely some sub-lineages with this targeted sequencing: for example, the Delta sub-lineage was not identified in 2 patients, and we could not discriminate between the 2 strains, Omicron variants 22A_BA.4 and 22B_BA.5, in 1 co-infected patient using the Spike amplicon. However, we did find multiple haplotypes in other co-infected patients and this haplotyping approach enabled us to precisely monitor the SARS-CoV-2 quasispecies, especially in a chronically infected patient on monoclonal antibodies whose virus developed resistance mutations. The patient got a superinfection, several haplotypes coexisted and 1 of his recombinant haplotypes was finally selected. As we found different viral haplotypes in 2 samples of this patient taken on the same day, his quasispecies probably evolved in an even more complex manner. We also identified minor resistance-associated mutations that had not been detected by whole genome sequencing. Targeted sequencing for haplotyping and complete genome analyses are therefore complementary.

Recombinant viruses were identified in 4 of the 17 co-infected patients, which is more frequent than the rate previously reported by Bolze et al. (1 Delta/Omicron 21K recombinant in 18 co-infections [20]) or Bal et al. (3 Delta/Omicron 21K and 1 21K/21L recombinant in 58 co-infections [13]). The prolonged infection with great virus loads, in the 2 of our 4 patients who were immunocompromised, might have favored the occurrence of recombination. The other 2 patients are epidemiologically linked, which might explain that they have several similar haplotypes. Previous studies have shown that transmission bottlenecks often limited the infection to 1 or a few viral particles (26–28). In this case and in the absence of sequential samples, we could not determine whether each individual was first infected by a single strain and then contaminated each other, or if several strains were transmitted simultaneously from one individual to the other, or if multiple transmissions events occurred between those 2 individuals. To note, we could not exclude that minority haplotypes in Patient 12 and Patient 13 result from single nucleotide polymorphisms of the parental strains rather than recombination events, given their genetic closeness. These prevalence results should be confirmed on a larger data set to determine the recombination frequency and the contributory factors more precisely.

Recombination events in co-infected patients are fundamental if a recombinant strain is to spread throughout the general population. We identified 4 cases of infection with recombinant viruses whose parental strains were absent. Although one of them does not have an attributed Pangolin lineage name, a similar mutation profile has been observed in other individuals without an epidemiological link; transmission of the recombinant strain therefore appears to be more plausible than co-infection followed by recombination in this individual. The frequency of direct infection with recombinant SARS-CoV-2 was about 0.06%, but this will depend on the fitness of each circulating recombinant strain. For example, the recombinant lineage XBB probably arose in mid-2022 and its subsequent expansion justified the creation of a new clade, 22F.

Several precautions were implemented to avoid the detection of “false” co-infections. First, to eliminate the cross-contamination, we confirmed all cases by carrying out, from the primary samples, a second nucleic acids extraction, amplification, and sequencing. This risk was also limited by the barcoding step that was performed early in the amplification protocol. To note, long amplicons (1.2 kb and 2.5 kb in our protocols) are less prone to contaminate the environment than shorter ones, such as those of the ARTIC protocol. Then, PCR and sequencing errors could also result in minority variants but the raw error rate of third generation sequencing techniques have much improved since their release (29). For example, we obtained here with SMRT sequencing a median Phred score of Q37 (accuracy: 99.98%). In addition, we confirmed co-infections by using two sequencing assays – whole genome and targeted Spike sequencing - on the 2 nucleic acid extracts. It was therefore highly improbable to observe random errors repeatedly at the same positions, and specifically at the clade/lineage-defining positions.

Long-read sequencing and haplotyping provide complementary information to the more commonly used short-read sequencing. They appear to facilitate the detection of co-infections and recombinant virus strains and contribute to a better understanding of SARS-CoV-2 genetic evolution.

## MATERIALS AND METHODS

### Patients and samples.

We sequenced SARS-CoV-2-positive samples taken in January-October 2022 to investigate severe cases of hospitalized or immunocompromised patients, clusters, 3-dose vaccination failures, and epidemiological monitoring. We first sequenced whole genomes and then the S genes (region encoding the Spike protein S1 domain) to confirm co-infections. We obtained SARS-CoV-2 sequences for 6829 samples collected from 6411 individuals: patients (*n* = 1644 samples) and staff (*n* = 422 samples) of Toulouse University Hospital (South-West France), people coming to our COVID-19 testing center (*n* = 4022 samples), and from other regional hospitals and laboratories (*n* = 741 samples).

### SARS-CoV-2 detection and VOC screening.

SARS-CoV-2 was detected using the MGIEasy Nucleic Acid Extraction kit on a MGISP-960 system (Beijing Genome Institute) to extract virus RNA, followed by an RT-PCR with the TaqPath COVID-19 CE-IVD RT-PCR kit (ThermoFisher Scientific) running on the QuantStudio 5 System (Applied Biosystems), or the Aptima SARS-CoV-2 Transcription-Mediated Amplification (TMA) Assay on the Panther System (Hologic). The TaqPath assay targets 3 viral genes including the S gene, whose detection is hampered in the case of del69/70. We initially used this characteristic of SGTF/SGTL detection for variant screening, to discriminate the first Omicron VOC 21K/BA.1 from the Delta VOC (30, 31). We later screened for variant-specific mutations using the ID SARS-CoV-2/VOC Revolution Pentaplex assay (ID-Solutions) after viral RNA extraction on the MGISP-960 system.

### SARS-CoV-2 whole genome sequencing.

Virus full-length sequences were obtained using a PacBio HiFiViral for SARS-CoV-2 workflow, based on 29 overlapping 1.2 kb amplicons, as previously described (17). Briefly, viral RNA was extracted with the MGISP-960 system and reverse transcribed using random hexamers and the Superscript IV VILO enzyme (Life Technologies). Two multiplex PCRs (1 of 15 and the other of 14 amplicons) were then performed using M13-tailed primers and the Q5 Hot Start High Fidelity DNA polymerase (New England Biolabs). The Midnight primers (32) we used had been adapted to cope with the genetic evolution of SARS-CoV-2. The second PCR used barcoded M13 primers and the Kapa HiFi HotStart ReadyMix (Roche Diagnostics) and 96 to 288 samples were multiplexed in each sequencing reaction. Libraries were sequenced using the SMRTbell Express Template Prep 2.0 kits, on a Sequel IIe instrument (PacBio), according to the manufacturer’s instructions.

The whole genome sequences were built from the PacBio reads using a custom-made Snakemake pipeline. The HiFi reads were directly generated by the Sequel IIe sequencer, then demultiplexed and filtered (minimum of 3 passes, Q20) with Lima (v2.0.0 to v.2.6.0, https://github.com/PacificBiosciences/barcoding). The resulting reads were analyzed by pbAA (v0.1.3, https://github.com/PacificBiosciences/pbAA), CoSA (Coronavirus Sequence Analysis, v9.0.0, https://github.com/Magdoll/CoSA), and mapped to the SARS-CoV-2 reference genome (Wuhan-Hu-1 isolate, GenBank accession number NC_045512.2) with Minimap2 (v2.17) to construct consensus sequences.

Lineages and clades were attributed according to the most recent versions of Pangolin (v3.1.17 to v.4.0.6 [2]) and NextClade (v1.8.0 to v2.8.0 [1]). Lists of mutations were extracted from Nextclade reports and their frequencies estimated with a PacBio variant caller tool - Juliet (v1.12.0, https://github.com/PacificBiosciences/minorseq).

### S gene sequencing.

We sequenced a 2490 nucleotide amplicon covering the full-length of the Spike protein S1 domain using the two A6 amplicon primers designed in the Pacific Biosciences protocol for full genome sequencing, as previously described (18) (https://www.pacb.com/wp-content/uploads/Procedure-Checklist-Multiplexing-2.5-kb-Amplicons-for-Whole-Genome-Sequencing-of-SARS-CoV-2.pdf, accessed on 30 June 2020). This A6 amplicon was generated using M13-tailed primers and the Platinum SuperFi Master Mix (Life Technologies) from the same cDNA as the SARS-CoV-2 whole genome described above. The second PCR used barcoded M13 primers and the Kapa HiFi HotStart ReadyMix (Roche Diagnostics). Libraries were prepared and sequenced as described above for the complete genome.

The HiFi reads were demultiplexed and filtered with Lima. The resulting reads were analyzed by pbAA (v1.0.2, https://github.com/PacificBiosciences/pbAA), which clustered similar reads together and generated 1 or more haplotypes, depending on the diversity of the viral population in each sample. Haplotype sequences were aligned to the reference SARS-CoV-2 spike region (NC_045512.2) with MAFFT (v.7.475 to v.7.505) (33) and trimmed from the beginning of the S gene (nt 21563) to the end of the A6 amplicon (nt 23823). Lists of mutations for each haplotype were extracted from Nextclade reports and their frequencies estimated with a PacBio variant caller tool - Juliet (v1.12.0, https://github.com/PacificBiosciences/minorseq).

### Statistics.

Median ages were compared between co-infected individuals and other SARS-CoV-2-infected individuals using a Wilcoxon test. The percentage of male/female in these 2 groups were compared using a Chi-square test.

### Data availability.

SARS-CoV-2 sequences were deposited on the GISAID database, except in cases of insufficient coverage (< 70% of the full-length genome) or not previously reported frameshifts; cf. Tables 2 and 3.
