# Supplementary material for: SARS-CoV-2 Co-Infections and Recombinations Identified by Long-Read Single-Molecule Real-Time Sequencing
Source: Microbiol Spectr. 2023 Jun 1;11(4):e00493-23. doi: 10.1128/spectrum.00493-23 (PMC10434069; doi:10.1128/spectrum.00493-23)
Supplement: Supplemental file 1 — Figures S1 and S2. Download spectrum.00493-23-s0001.pdf, PDF file, 1.6 MB [file spectrum.00493-23-s0001.pdf]

Supplementary data

Patient 1.

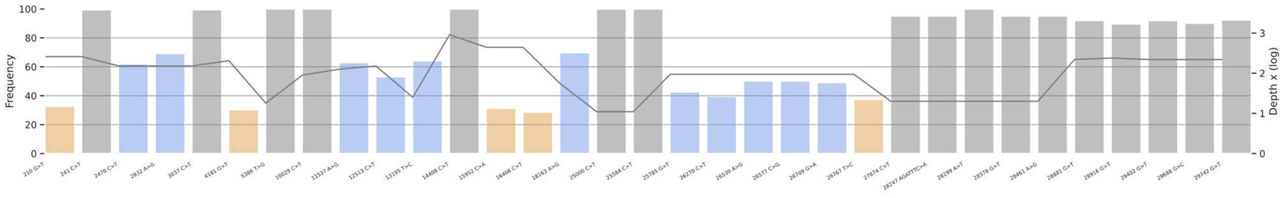

Patient 2.

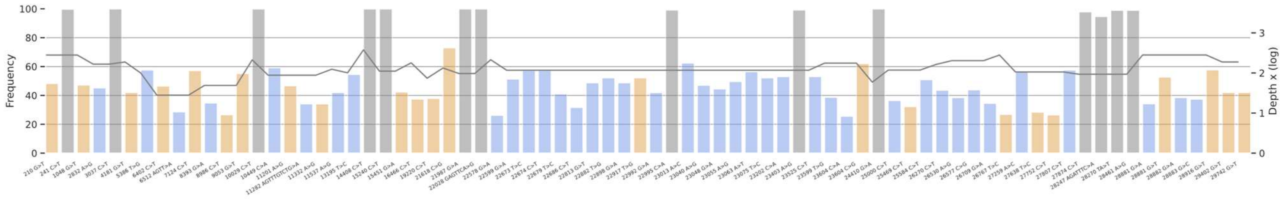

Patient 3.

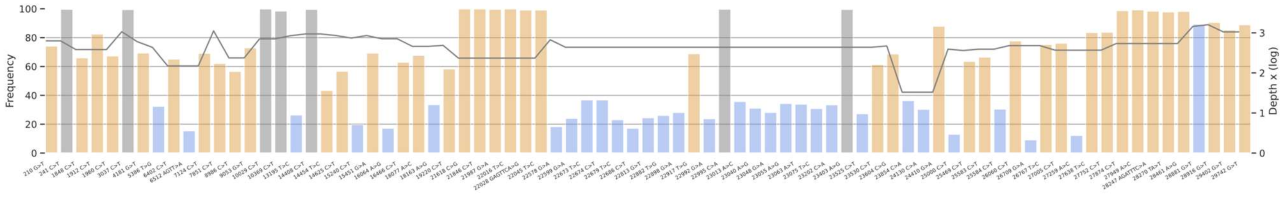

Patient 4.

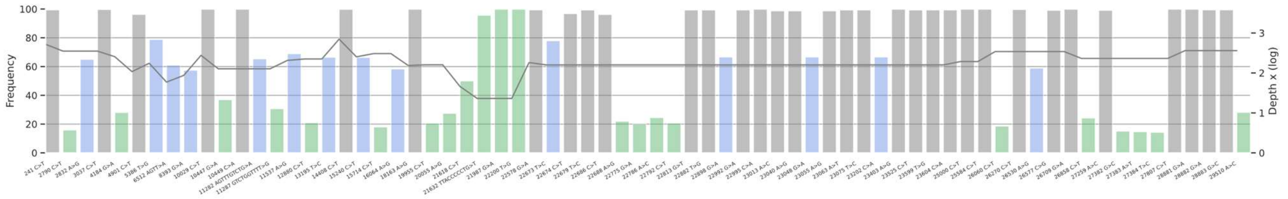

Patient 5.

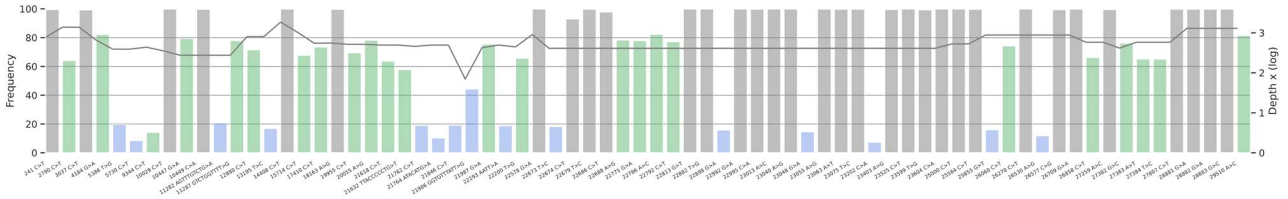

Patient 6.

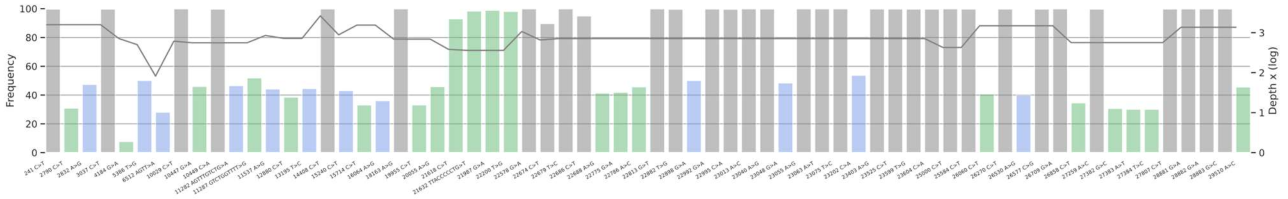

Patient 7 (sample 7e).

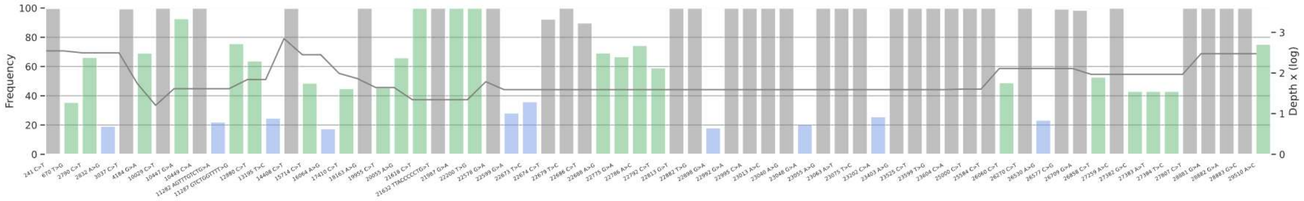

Patient 8 (sample 8b).

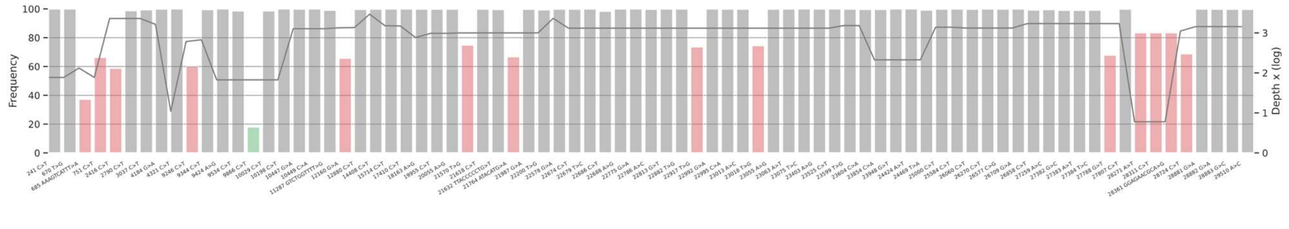

Patient 9.

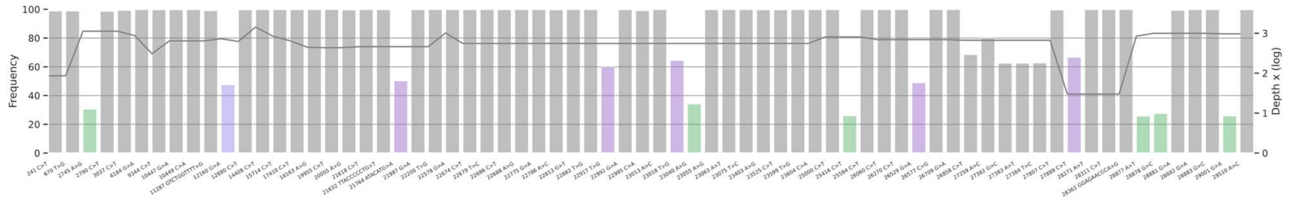

Patient 10.

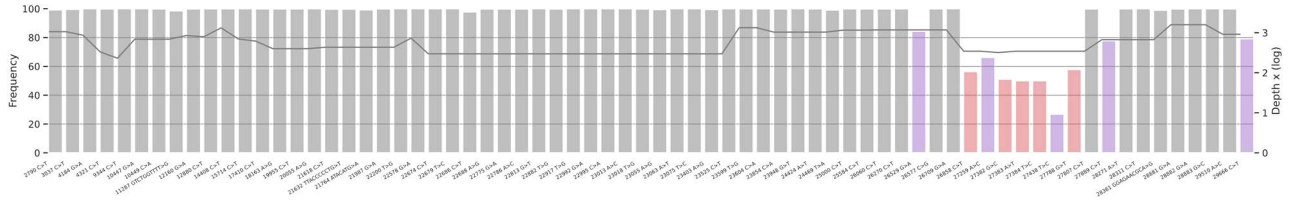

Patient 11.

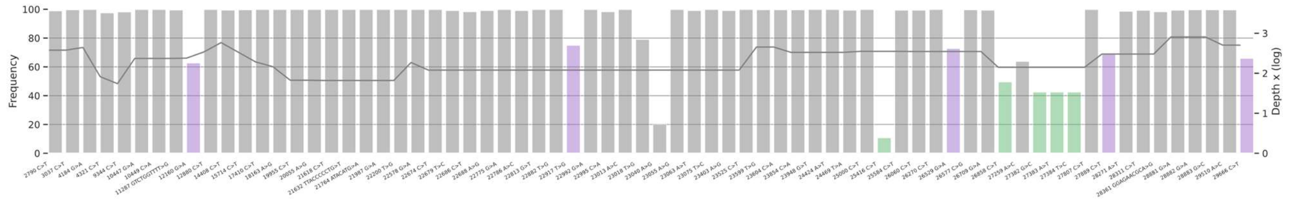

Patient 12.

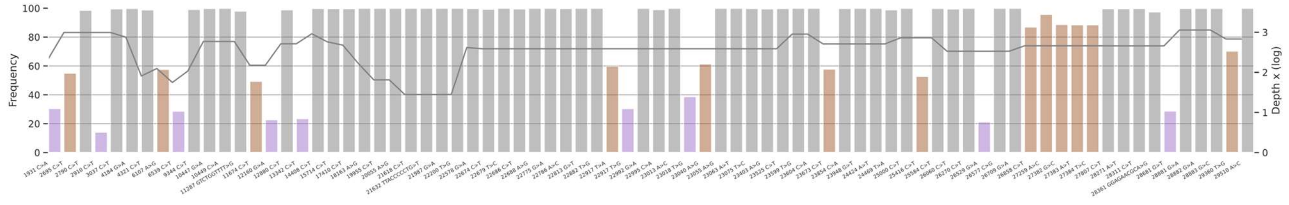

Patient 13.

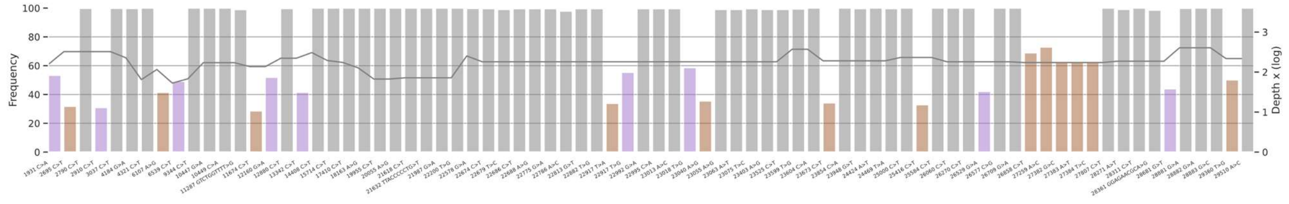

Patient 14.

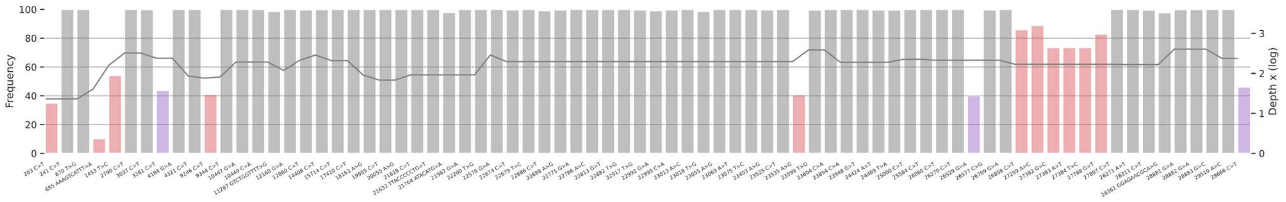

Patient 15.

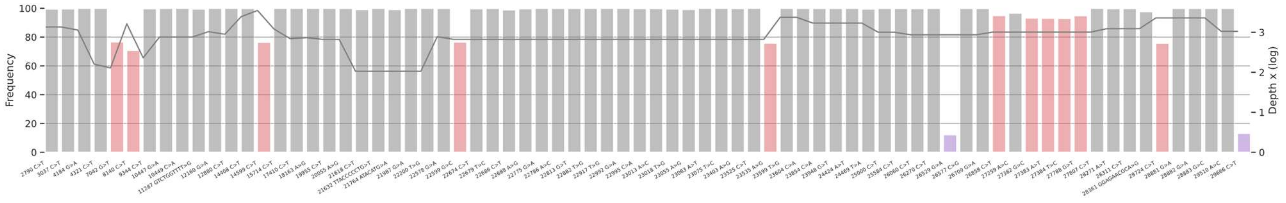

Patient 16.

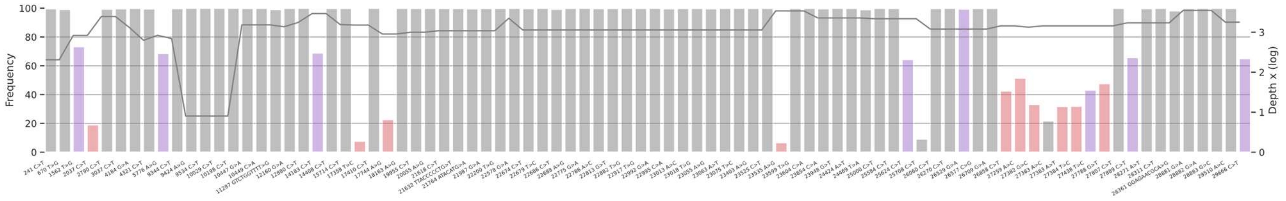

Patient 17.

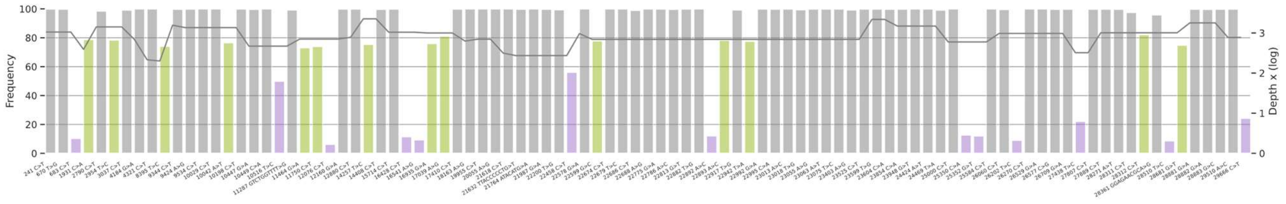

Figure S1. Nucleotide mutation frequencies in all co-infection cases

Each panel displays the relative abundance of mutations compared to the Wuhan reference sequence for one co-infected patient (left axis). Grey bars represent polymorphisms common to both infecting SARS-CoV-2 lineages and colored bars represent clade/lineage-defining polymorphisms (yellow: 21A/I/J_B.1.617.2, blue: 21K_BA.1, dark green: 21L_BA.2, red: 22A_BA.4, purple: 22B_BA.5, maroon: 22C_BA.2.12.1, light green: 22E_BQ.1). Black lines indicate the coverage depth (right axis).

Patient 7.

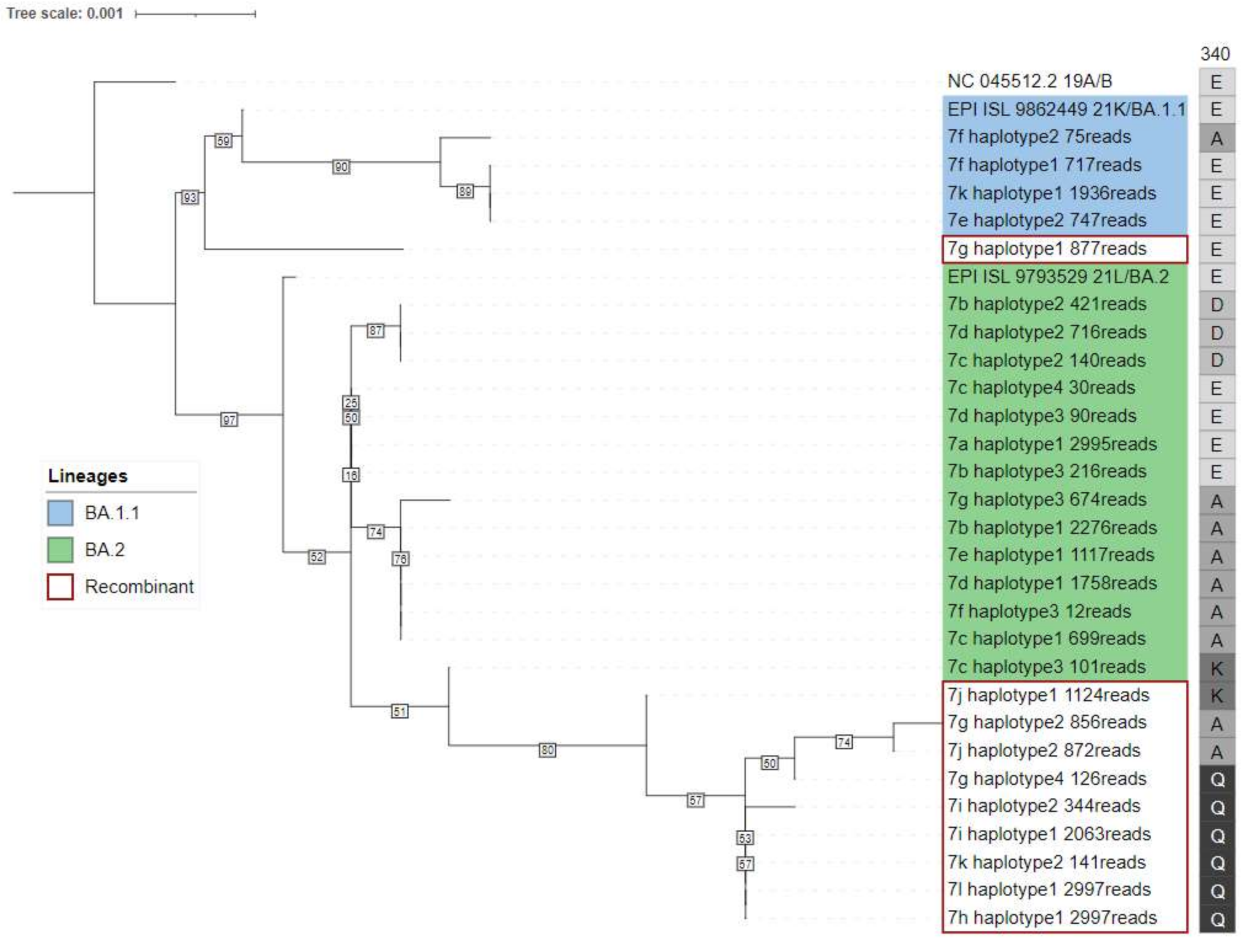

Patient 8.

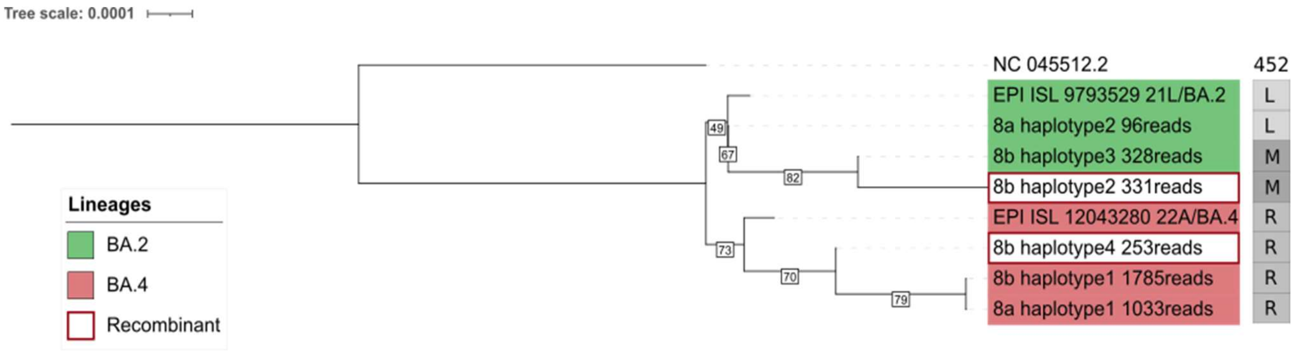

Figure S2. Evolution of Spike amplicon haplotypes in two immunocompromised individuals

Phylogenetic trees were constructed using IQTREE (v.2.0.3, model: GTR+F+R3) from MAFFT multiple alignments. The trees were rooted on the Wuhan reference (NC 045512.2). All Spike amplicon haplotypes from each individual were included in the phylogenetic analyses (genome positions of the amplicon: 21563-23823). Bootstrap values are written on the branches (ultrafast bootstrap: 1000 replicates).

Patient 7: samples were collected from Patient 7 between February, 3rd 2022 (samples 7a) and March, 28th 2022. Annotations on the right-hand side of the tree indicates the amino-acid at position 340 of the Spike protein (frequent mutations under Sotrovimab selective pressure).

Patient 8: two samples were collected one week apart. Annotations on the right-hand side of the tree indicates the amino-acid at position 452 of the Spike protein.
